# Association of Serum Level of 25-Hydroxy Vitamin D Deficiency and Pulmonary Function in Healthy Individuals

**DOI:** 10.1155/2018/3860921

**Published:** 2018-04-26

**Authors:** Maryam Moghaddassi, Marzieh Pazoki, Ahmad Salimzadeh, Tayeb Ramim, Zahra Alipour

**Affiliations:** ^1^Rheumatology Research Center, Sina Hospital, Tehran University of Medical Sciences, Tehran, Iran; ^2^Sina Hospital, Tehran University of Medical Sciences, Tehran, Iran; ^3^Cancer Pharmacogenetics Research Group (CPGRG), Iran University of Medical Sciences, Tehran, Iran; ^4^Research Development Center, Sina Hospital, Tehran University of Medical Sciences, Tehran, Iran

## Abstract

**Background:**

Besides the extensive regulatory role in growing number of biologic processes, vitamin D has been recently considered essential for lungs function as well as protective against exacerbation of chronic obstructive pulmonary diseases. We assessed the correlation between vitamin D serum levels with pulmonary function in healthy individuals.

**Methods:**

In a cross-sectional study, healthy volunteer (*n* = 92) participants underwent the following laboratory procedures: a blood test, a 24-hour urine collection test, and the serum level of 25-hydroxy vitamin D before undergoing spirometry. Linear correlation coefficient was calculated to detect the association between serum level of 25-hydroxy vitamin D and pulmonary volumes.

**Results:**

The mean age of participants was 39.95 ± 9.98 years. 48% of participants showed different levels of 25-hydroxy vitamin D deficiency. We recognized a consistent direct positive correlation between serum levels of 25-hydroxy vitamin D and lung function volumes. The coefficient for forced vital capacity, forced expiratory volume in 1 second, forced expiratory flow 25–75%, and forced expiratory volume in 1 second/forced vital capacity ratio were 0.610, 0.509, 0.454, and 0.551, respectively.

**Conclusions:**

Our findings suggest correlation between higher serum levels of 25-hydroxy vitamin D and improved pulmonary function. Accordingly, supplemental vitamin D might significantly improve treatment response.

## 1. Introduction

Vitamin D is a fat-soluble vitamin extensively being considered for its essential role in calcium homeostasis and bone formation. The serum level of vitamin D is a regulated by exposure to the sunlight as well as dietary intake [1, 2]. Vitamin D, however, has attracted remarkable attention for its role in diverse biologic processes during past years, including antiproliferative and antiapoptotic effect [3–5], prevention from cardiovascular diseases [2, 3], modulating immunity [4, 5], preventing autoimmune diseases (multiple sclerosis, diabetes, rheumatoid arthritis, systemic lupus erythematosus, etc.) [5–9], and expression of antimicrobial peptides and receptors [10–14]. The role of vitamin D in the respiratory infections prevention and treatment (particularly tuberculosis) has already been well-established [10–13]. The list of suggested roles for vitamin D is still growing; for example, studies have been linking psychosis to vitamin D deficiency [15, 16].

Recent studies have proposed a relationship between serum vitamin D level and the lungs function in both adult and children [17–19]. Moreover, papers have indicated that consumption of vitamin D supplements could protect against moderate to severe exacerbation of chronic obstructive pulmonary diseases (COPD) [20, 21], whereas some researchers believe that those beneficial effects have been demonstrated in vitro rather than in vivo and real life reports have been conflicting [22].

Despite the global prevalence of vitamin D deficiency [23–25] and the reported correlation of vitamin D level and COPD, only few studies have investigated association between pulmonary function test in healthy individuals and vitamin D level. Such study can better improve our understanding of the controversy regarding protective effects of vitamin D on the pulmonary system. This study is to evaluate the correlation between vitamin D serum levels with pulmonary volumes in healthy individuals.

## 2. Materials and Methods

*Study Population*. In this cross-sectional study, we recruited healthy volunteers with negative history of pulmonary, cardiovascular, and muscular diseases who responded to a posted advertisement during a one-year study period. The exclusion criteria were receiving therapeutic dosage of vitamin D during the preceding 6 months, respiratory tract infections (either lower or upper, regardless of being viral or bacterial) during 8 weeks prior to the study, administration of bronchodilators (beta-agonists, anticholinergics, inhaled and systemic corticosteroids, and methylxanthines) within 7 days before the study, and smoking ≥ 20 pack-year.

Demographic data, including age, gender, height, weight, and body mass index (BMI), were recorded for all enrolled participants. Participants were asked to undergo laboratory procedures that comprised (1) a blood work: 10 milliliter of brachial venous blood was sampled for calcium, phosphorus, alkaline phosphatase, 25-hydroxy vitamin D (25(OH)D), blood urea nitrogen (BUN), creatinine (Cr), and parathyroid hormone (parathormone), and (2) a 24-hour urine collection test for calcium, phosphorus, creatinine, and urine volume. The serum level of 25(OH)D was assessed by enzyme-linked immunosorbent assay (ELISA) method. After laboratory procedures, all participants underwent spirometry for forced vital capacity (FVC), forced expiratory volume in 1 second (FEV1), FEV1/FVC ratio, and forced expiratory flow 25–75% (maximum mid-expiratory flow, FEF_25–75%_). Pulmonary function test measured the amount of air that participant breathes in and out. The person sat in front of a spirometer and was fitted with a mouthpiece and wore a nose clip so that all the air goes into the spirometer. The technologist asked the person to breathe in and out as deeply or as quickly as they can for several seconds. One expert technologist measured the pulmonary function in present study.

Cochran's sample size formula was used in alpha error = 5% and Beta error = 20% and sample power was 80%.

*Ethics Approval and Consent*. The protocol of the present study was approved by Tehran University of Medical Sciences under registered number 16419 in 2012. Bioethics committee of the Tehran University of Medical Sciences approved the study protocol. Participants' consent was obtained through signing a written informed consent form.

*Statistical Analysis*. For qualitative variables, absolute and relative frequency, and, for quantitative variables, the mean with two standard deviations were calculated. Linear correlation coefficient was calculated to detect the association between serum level of 25(OH)D and pulmonary volumes. The analysis was conducted with SPSS ver. 19 (IBM Corp., USA) and *P* value < 0.05 was considered statistically significant.

## 3. Results

Thirty subjects out of ninety-two enrolled participant were male (32.60%). The mean age of participants (spanned 25 to 60 years) was 39.95 (±9.98) years. Age distribution of participants was shown in Table 1.

The mean of participants' height and weight was 165.64 (±7.02) cm and 68.2 (±9.51) kg, respectively. Accordingly, the mean BMI was 24.29 (±3.85) kg/m^2^, ranging from 16 to 32 kg/m^2^. The results of participants' blood and 24-hour urine samples are summarized in Table 2. No calcium and phosphorus disturbance was detected, nor was renal insufficiency diagnosed.  Table 3 represents serum levels of 25(OH) D of the participants matched with reference values.  Table 4 delineates the results of participants' spirometry in terms of four measured indices.

**Figure 1:**
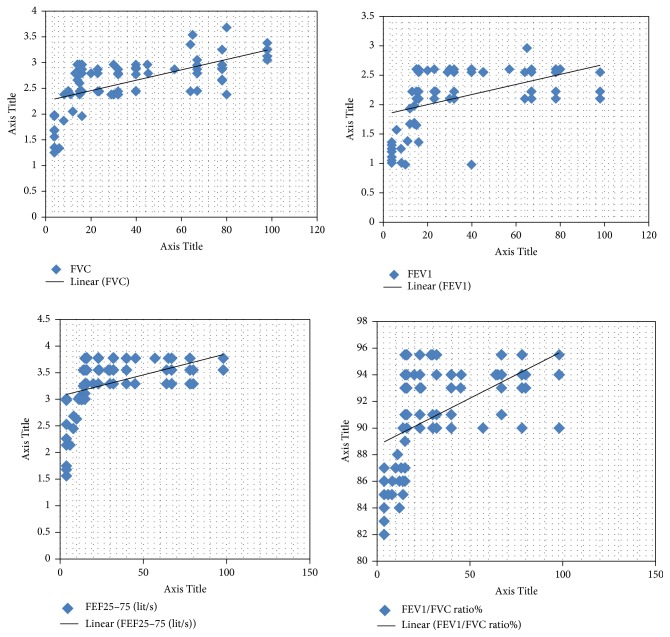
Positive correlation of the serum 25(OH)D with indices of spirometry test in healthy individuals. FVC, forced vital capacity; FEV1, forced expiratory volume in 1 second; FEF25–75%, maximum mid-expiratory flow or forced expiratory flow 25–75%.

Calculating linear correlation coefficient for association between serum level of 25(OH) D and pulmonary volumes indicated a consistent direct positive correlation between serum levels of 25(OH)D and lung function volumes. The coefficients for FVC, FEV1, FEF_25–75%_, and FEV1/FVC ratio were 0.610, 0.509, 0.454, and 0.551, respectively.  Figure 1 depicts such a correlation for all four indices. Also, after adjusting for age, gender, and BMI, the results showed correlations for FVC, FEV1, FEF_25–75%_, and FEV1/FVC ratio were 0.654, 0.515, 0.615, and 0.612, respectively.

## 4. Discussion

The authors of this study recruited only healthy individuals to shed light on the preventive role of vitamin D and its baseline status. The results of the present study emphasize a positive correlation between the serum level of 25(OH)D and pulmonary function. Although relationship of vitamin D with respiratory tract infections has already been studied, its association with normal pulmonary volumes has not been under the spotlight so far.

In one of such few studies, Palacios and Gonzalez performed spirometry on 14,000 people over 20 years old and postulated a strong relationship between the average serum concentrations of 25(OH)D and the average FEV1 and FVC volumes (*P* < 0.001) [24]. This finding is consistent with our finding in the present study.

Other studies have concluded that decreased serum level of vitamin D has been associated with pulmonary dysfunction, increased airway hyperresponsiveness, and reduced therapeutic response to glucocorticoids suggesting that supplemental vitamin D for an improved treatment response would be likely justifiable [26]. This conclusion is in accordance with our finding that higher serum vitamin D levels correlate with healthier respiratory volumes. Sutherland and colleagues pointed out that for every one nanogram/milliliter increase in vitamin D serum concentration, FEV1 would experience 22.7 (±9.3) milliliter increments. In another study, a 6-month follow-up of 25(OH)D baseline in 800 Finnish young adults revealed that serum 25(OH)D level lower than 40 nanomol/liter was significantly associated with more sick leaves due to respiratory tract infections (*P* = 0.004) [27]. Li et al. found that low serum vitamin D was associated with lower lung function in Chinese asthma patients [28].

The mean of serum 25(OH)D level in the aforementioned study was 80.2 (±29.3) nanomol/liter. In addition, the discrepancy of 25(OH)D serum level between smoker cases and nonsmoker controls was statistically significant (*P* < 0.001).

In our study, we excluded smokers in order to evaluate the net effect of serum vitamin D on lung volumes; though this rendered comparison between smokers and nonsmokers impossible. The authors recommend further investigations to determine if serum 25(OH)D levels in healthy individuals is of prognostic value for upcoming pulmonary/respiratory disorders. We also recommend studying lung volumes in people with vitamin D deficiency in order to help better understand the impact of vitamin D on respiratory tract.

## 5. Conclusions

The results of our study indicate that higher serum levels of 25-hydroxy vitamin D correlate with improved pulmonary function capabilities. Therefore vitamin D supplementation may be useful for improving pulmonary function capabilities.

## Figures and Tables

**Table 1:** Age distribution of healthy participants.

Age group (year)	*N* (%)
25–30	5 (5.40)
30–35	11 (11.90)
35–40	25 (27.17)
40–45	23 (25.00)
45–50	18 (19.56)
50–55	6 (6.52)
55–60	1 (4.34)

**Table 2:** Healthy participants' blood and 24-hour urine samples studied for association between serum 25(OH)D and pulmonary volumes.

Variable	Mean (±SD^*∗*^)	Normal reference
*Blood chemistry*		
BUN^†^	24.67 (±3.20)	18–55 (mg/dl)^‡^
Creatinine	0.80 (±0.19)	0.7–1.4 (mg/dl)
Calcium	8.70 (±0.23)	8.5–10.2 (mg/dl)
Phosphorus	3.40 (±0.60)	2.4–4.5 (mg/dl)
Alkaline phosphatase	74 (±11.04)	35–100 (U/L)^§^
Parathyroid hormone	32.22 (±13.28)	11–54 (pg/ml)^¶^
*24-hour urine analysis*		
Total volume	1772.83 (±476.97)	800–2000 (ml/24 h)
Creatinine	1122.11 (±412.05)	500–2000 (mg/24 h)
Calcium	136.34 (±96.01)	100–250 (mg/24 h)
Phosphorus	545.40 (±111.25)	400–1300 (mg/24 h)

^*∗*^SD, standard deviation; ^†^BUN, blood urea nitrogen; ^‡^mg/dl, milligram per deciliter; ^§^U/L, unit per liter; ^¶^pg/ml, picogram per milliliter.

**Table 3:** Distribution of the matched levels of 25(OH)D for the studied healthy individuals.

Variable	Number (%)	Normal reference (ng/ml)^*∗*^
25(OH)D		
Sufficient	48 (52.20)	30–100
Insufficient	8 (8.70)	20–30
Deficient	25 (27.20)	10–20
Very deficient	11 (12.00)	<10

^*∗*^ng/ml, nanogram per milliliter.

**Table 4:** The results of healthy participants' spirometry tests.

Lung function index	Measured lung function (mean ± SD)	Predicted lung function (%)
FVC^*∗*^ (Lit)	2.60 (±0.47)	88.27 (2.41)
FEV1^†^ (Lit)	2.10 (±0.51)	89.41 (1.05)
FEF_25–75%_^‡^ (Lit/S)	3.33 (±0.55)	106.64 (3.71)
FEV1/FVC	91.41 (±3.64)	112.87 (5.89)

^*∗*^FVC, forced vital capacity; ^†^FEV1, forced expiratory volume in 1 second; ^‡^FEF25–75%, maximum mid-expiratory flow or forced expiratory flow 25–75%. All four spirometry indices were measured as liter (Lit), except for forced expiratory flow (FEF) that was measured as liter per second (Lit/S) and represented as mean (±SD).

## References

[1] Das G., Crocombe S., McGrath M., Berry J. L., Mughal M. Z. (2005). Hypovitaminosis D among healthy adolescent girls attending an inner city school. *Archives of Disease in Childhood*.

[2] Heine G., Lahl A., Müller C., Worm M. (2010). Vitamin D deficiency in patients with cutaneous lupus erythematosus is prevalent throughout the year. *British Journal of Dermatology*.

[3] Moreno J., Krishnan A. V., Feldman D. (2005). Molecular mechanisms mediating the anti-proliferative effects of Vitamin D in prostate cancer. *The Journal of Steroid Biochemistry and Molecular Biology*.

[4] Raiten D. J., Picciano M. F. (2004). Vitamin D and health in the 21st century: bone and beyond. Executive summary. *American Journal of Clinical Nutrition*.

[5] Holick M. F. (2004). Sunlight and vitamin D for bone health and prevention of autoimmune diseases, cancers, and cardiovascular disease. *American Journal of Clinical Nutrition*.

[6] Munger K. L., Levin L. I., Hollis B. W., Howard N. S., Ascherio A. (2006). Serum 25-hydroxyvitamin D levels and risk of multiple sclerosis. *Journal of the American Medical Association*.

[7] Littorin B., Blom P., Schölin A. (2006). Lower levels of plasma 25-hydroxyvitamin D among young adults at diagnosis of autoimmune type 1 diabetes compared with control subjects: Results from the nationwide Diabetes Incidence Study in Sweden (DISS). *Diabetologia*.

[8] Merlino L. A., Curtis J., Mikuls T. R., Cerhan J. R., Criswell L. A., Saag K. G. (2004). Vitamin D intake is inversely associated with rheumatoid arthritis: results from the Iowa Women’s Health Study. *Arthritis & Rheumatism*.

[9] Yap K. S., Northcott M., Hoi A. B.-Y., Morand E. F., Nikpour M. (2015). Association of low Vitamin D with high disease activity in an Australian systemic lupus erythematosus cohort. *Lupus Science & Medicine*.

[10] Wang T.-T., Nestel F. P., Bourdeau V. (2004). Cutting edge: 1,25-Dihydroxyvitamin D3 is a direct inducer of antimicrobial peptide gene expression. *The Journal of Immunology*.

[11] Gombart A. F., Borregaard N., Koeffler H. P. (2005). Human cathelicidin antimicrobial peptide (CAMP) gene is a direct target of the vitamin D receptor and is strongly up-regulated in myeloid cells by 1,25-dihydroxyvitamin D_3_. *The FASEB Journal*.

[12] Liu P. T., Stenger S., Li H. (2006). Toll-like receptor triggering of a vitamin D-mediated human antimicrobial response. *Science*.

[13] Coussens A. K., Wilkinson R. J., Hanifa Y. (2012). Vitamin D accelerates resolution of inflammatory responses during tuberculosis treatment. *Proceedings of the National Academy of Sciences of the United States of America*.

[14] Holick M. F. (2005). The vitamin D epidemic and its health consequences. *Journal of Nutrition*.

[15] McGrath J., Eyles D., Mowry B., Yolken R., Buka S. (2003). Low maternal vitamin D as a risk factor for schizophrenia: A pilot study using banked sera. *Schizophrenia Research*.

[16] Lally J., Gardner-Sood P., Firdosi M. (2016). Clinical correlates of vitamin D deficiency in established psychosis. *BMC Psychiatry*.

[17] Shaheen S. O., Jameson K. A., Robinson S. M. (2011). Relationship of vitamin D status to adult lung function and COPD. *Thorax*.

[18] Yao T. C., Tu Y. L., Chang S. W. (2014). Serum 25-hydroxyvitamin D levels in relation to lung function and exhaled nitric oxide in children. *Journal of Pediatrics*.

[19] Mekov E., Slavova Y., Tsakova A. (2015). Vitamin D deficiency and insufficiency in hospitalized COPD patients. *PLoS ONE*.

[20] Martineau A. R., James W. Y., Hooper R. L. (2015). Vitamin D3 supplementation in patients with chronic obstructive pulmonary disease (ViDiCO): a multicentre, double-blind, randomised controlled trial. *The Lancet Respiratory Medicine*.

[21] Lehouck A., Mathieu C., Carremans C. (2012). High doses of vitamin D to reduce exacerbations in chronic obstructive pulmonary disease. *Annals of Internal Medicine*.

[22] Janssens W., Decramer M., Mathieu C., Korf H. (2013). Vitamin D and chronic obstructive pulmonary disease: hype or reality?. *The Lancet Respiratory Medicine*.

[23] Mithal A., Wahl D. A., Bonjour J. P. (2009). Global vitamin D status and determinants of hypovitaminosis D. *Osteoporosis International*.

[24] Palacios C., Gonzalez L. (2014). Is vitamin D deficiency a major global public health problem?. *The Journal of Steroid Biochemistry and Molecular Biology*.

[25] Calvo M. S., Whiting S. J., Barton C. N. (2005). Vitamin D intake: a global perspective of current status. *Journal of Nutrition*.

[26] Sutherland E. R., Goleva E., Jackson L. P., Stevens A. D., Leung D. Y. M. (2010). Vitamin D levels, lung function, and steroid response in adult asthma. *American Journal of Respiratory and Critical Care Medicine*.

[27] Laaksi I., Ruohola J. P., Tuohimaa P. (2007). An association of serum vitamin D concentrations <40 nmol/L with acute respiratory tract infection in young Finnish men. *The American Journal of Clinical Nutrition*.

[28] Li F., Peng M., Jiang L. (2011). Vitamin D deficiency is associated with decreased lung function in Chinese adults with asthma. *Respiration*.

